# Caries-associated oral microbiome in head and neck cancer radiation patients: a longitudinal study

**DOI:** 10.1080/20002297.2019.1586421

**Published:** 2019-03-08

**Authors:** Jean-Luc C. Mougeot, Craig B. Stevens, Kathryn G. Almon, Bruce J. Paster, Rajesh V. Lalla, Michael T. Brennan, Farah Bahrani Mougeot

**Affiliations:** a Carolinas Medical Center - Atrium Health, Charlotte, NC, USA; b The Forsyth Institute, Cambridge, MA, USA; c University of Connecticut Health, Farmington, CT, USA

**Keywords:** Head and neck cancer, radiation therapy, caries, microbiome, 16S rRNA, next generation sequencing

## Abstract

Head and neck cancer (HNC) therapy often leads to caries development. Our goal was to characterize the oral microbiome of HNC patients who underwent radiation therapy (RT) at baseline (T0), and 6 (T6) and 18 (T18) months post-RT, and to determine if there was a relationship with increased caries. HOMI*NGS* was used to determine the relative abundance (RA) of >600 bacterial species in oral samples of 31 HNC patients. The DMFS score was used to define patient groups with tooth decay increase (DMFS[+]) or no increase (DMFS[-]).A change in microbiome beta-diversity was observed at T6 and T18. The *Streptococcus mutans* RA increased at T6 in both DMFS[+] and DMFS[-] groups. The RA of *Prevotella melaninogenica*, the species often associated with caries in young children, decreased at T6 in the DMFS[-] group. The RA of the health-associated species, *Abiotrophia defective*, decreased in the DMFS[+] group. The oral microbiome underwent significant changes in radiation-treated HNC patients, whether they developed caries or not. Caries rates were not associated with a difference in salivary flow reduction between DMFS[+] andDMFS[-] groups. Patients who develop caries might be more susceptible to certain species associated with oral disease or have fewer potentially protective oral species.

Head and neck cancer (HNC) affects approximately 550,000 people worldwide, causing 380,000 deaths annually. With a five-year survival rate of 50–60%, men are affected roughly two to four times more than women [1,2]. HNC refers to tumors found primarily in the oral cavity, nasopharynx, oropharynx, hypopharynx, larynx and trachea, of which over 90% are squamous cell carcinomas (SCC) [3]. Major risk factors for HNC include tobacco use, excessive alcohol consumption, and the presence of human papilloma virus [4]. However, the occurrence of HNC in patients having none of these exposures suggests the presence of other causative risk factors [5]. The majority of HNC patients are treated with high-dose radiation therapy (RT), often in combination with chemotherapy and surgery [5,6]. Although these treatments improve patient survival, they often result in significant tissue damage, exposing the patient to chronic long-term complications including xerostomia, caries and osteoradionecrosis [6,7].

The central dogma regarding caries development in radiation-treated HNC patients is that salivary hypofunction is an important contributor when associated with a carbohydrate-rich diet that creates a microenvironment in dental plaque favorable to the growth of acidogenic and aciduric bacteria [6–14]. Prolonged increased levels of acid in plaque from bacterial metabolic activity can lead to enamel and dentin demineralization, ultimately resulting in the development of caries [15]. RT has been shown to cause direct damage to the salivary glands of HNC patients, often leading to hyposalivation [6,7]. In addition, radiation-induced disruption of enamel and dentin has been characterized *in vitro*, demonstrating its contribution to the weakening of tooth structure and increasing the potential for fracture [16].

A systematic review by Hong et al. showed an overall increase in prevalence of dental caries in chemotherapy and/or radiation-treated cancer patients compared to healthy controls, based on DMFS (decayed, missing and filled surface) scores [13]. The reasons why there are differences in the presence and severity of caries in response to the same level of RT have not yet been fully elucidated.

An association between dental demineralization, RT and post-RT dental caries has been identified in a few studies of HNC patients, but the involvement of specific oral microbiome profiles within saliva, dental plaque or other oral sites remains a matter of debate [17,18]. Thus, it is unclear whether specific microbiome profiles present in the oral cavity prior to cancer treatment constitute a higher risk for the development of caries in HNC patients undergoing RT.

Advancement in metagenomic 16S rRNA gene sequencing has allowed for identification and relative quantification of oral microbiome species [19]. A few metagenomic studies have investigated oral microbiome shifts in radiation-treated HNC patients, but none have identified specific bacterial profiles associated with the development of caries. In a study of eight HNC patients, Hu et al. used 16S rDNA pyrosequencing to determine microbial shifts in supragingival plaque before, and at several time points during RT [20]. That study analyzed the microbiome mostly at the genus level and did not investigate associations with the occurrence of post-RT dental caries. In another study using 16S rDNA sequencing, Zhang et al. conducted a cross-sectional analysis of saliva from a small number of adults with nasopharyngeal carcinoma, comparing those who developed caries (n = 9) to those who did not (n = 12) within 12 to 36 months post-RT [21]. This study determined changes in 11 genera, some of which contained caries-associated species, but a clear correlation between the salivary microbiota and the development of radiation caries was not established. The authors acknowledged the need for a more comprehensive longitudinal study to yield conclusive results.

To gain a more comprehensive understanding of the oral microbiome in the development of post-RT caries in HNC patients, we compared the oral microbiome profiles of HNC patients with SCC, based on16S rRNA analyses using Human Oral Microbe Identification using Next *G*eneration *S*equencing (HOMI*NGS*) [22]. We analyzed bacterial genomic DNA extracted from buccal mucosa, superficial supragingival plaque (SSP), tongue and saliva samples to determine microbiome relative abundance (RA) changes in HNC patients at baseline (prior to RT), and 6 and 18-months post-RT. In this study, we hypothesized that the changes in the oral microbiome profile will be different between 6- and 18-months following RT and that these changes will distinguish patients who develop caries compared to those who do not.

## Materials and methods

### Patient recruitment

HNC patients diagnosed with SCC (n = 31; 26 males, 5 females; mean age (SD) = 56.81 (10.74); age range = 24–84) were recruited to this study at Carolinas Medical Center (CMC)-Atrium Health and the University of Connecticut (UConn) Health Center within an ongoing longitudinal study on RT-associated clinical outcomes in HNC patients, namely ORARAD (U01-DE022939) [23,24]. The ORARAD study is a multicenter observational cohort study that collects data on dental outcomes in RT-treated HNC patients at 6-month intervals for two years. In the present study, however, oral samples were collected at baseline pre-RT (T0), post-RT 6-months (T6) and 18-months (T18). Oral samples were not collected at 12 months post-RT, although the DMFS score data were available. A detailed description of methodology and an extensive overall description of the ORARAD study population regarding primary tumor site, oral hygiene and dental disease measures, and other oral complications have been previously reported [23–25].

All patients in the T0 to T6 study (n = 31) received IMRT with a total dose ranging from 5,400 to 7,200 cGy (mean [SD] = 6,743,5 [442.2]) delivered as daily fractions over a period of 5–7 weeks. HNC patients received RT with or without concurrent chemotherapy (CC), with or without induction chemotherapy (IC), and with or without antibiotics (AB) treatment within 2 weeks prior to oral sampling. Patients had at least ten natural teeth pre- and post-RT. Oral hygiene recommendation included at least twice per day brushing, daily flossing and application of a prescription fluoride toothpaste daily. Patients were excluded if they were diagnosed with salivary gland malignancy or non-squamous cell carcinoma. This oral microbiome study was approved by IRB at both centers, for which all patients signed an informed consent (IRB #11–13-04A).

Demographics and clinical characteristics of all patients (Set-1, n = 31) are shown in Table 1. Less detailed clinical information for patients (Set-2n-22) having the same oral site samples at all three timepoints, paired T0 to T6 and T0 to T18 (*aka* T0 to Tx) is presented in Supplementary Table 1. T0 samples were taken within 6 weeks prior to RT, and T6 and T18 samples were taken within 30 days before or after the post-RT checkup date.

**Table 1.** Demographic and clinical data for patients with squamous cell carcinoma undergoing radiation therapy (Subset-A, n = 28 or 20).

Criteria	T0 to T6^a^			T0 to T18^b^		
**Total Pts (M/F)**	28 (23/5)			20 (16/4)		
**Age**						
Median	56			56		
Mean (SD)	56.2 (10.8)			56.3 (7.0)		
Range	24–84			40–70		
**Ethnicity^c^**						
**M** C/B/H	20/2/1			15/1/0		
**F** C/B/H	4/1/0			3/1/0		
	**DMFS[+]^d^**	**DMFS[-]**	p-value	**DMFS[+]**	**DMFS[-]**	p-value
**Total Pts (M/F)**	11 (10/1)	17 (13/4)	0.330*	7 (6/1)	13 (10/3)	0.639*
**Radiation Dose (cGy)**						
Median	7000	7000	0.746**	7000	6800	0.434**
Mean (SD)	6799 (419)	6721 (469)		6839 (286)	6500 (757)	
Range	6000–7200	5400–7020		6000–7020	5400–7000	
**DMFS (score) -T0/TX**						
Median	35/38	35/33		35/35.5	42/41	
Mean	39.2/42.9	39.1/38.5		35.8/37.6	51.5/50.3	
**SSFR (ml/min) -T0/TX**						
Median	0.97/0.23	0.99/0.29	0.452/0.370**	0.93/0.38	0.63/0.36	0.793/0.539**
Mean	1.41/0.21	1.16/0.35		1.16/0.49	1.00/0.36	

**^a^**T0 to T6 corresponds to baseline to 6-months post-RT sampling. **^b^**T0 to T18 corresponds to baseline to 18-months sampling. **^c^**Ethnicity: C (Caucasian), B (Black), H (Hispanic) and associated gender: Male (M), Female (F). DMFS is Decayed/Missing/Filled Surfaces scoring. **^d^**DMFS [+]: Patients whose DMFS score increased from T0 to T6 or T0 to T18. DMFS [-]: Patients whose DMFS score did not increase from T0 to T6 or T0 to T18. SSFR: stimulated salivary flow rate. SD is standard deviation. *Chi-squared test **Mann-Whitney U-test

Clinical information of patients in Set-1 subsets A, B, C, D for which DMFS score and stimulated salivary flow rate (SSFR) information is provided in Supplementary Table 2. These consist of Subset A (n = 24), all patients; Subset B (n = 20), patients who did not receive IC; Subset C (n = 21), patients who did not receive AB; and Subset D (n = 24), patients who received CC. DMFS score information collected at T0, T6, and T18 was available for 28 of the 31 patients and was used to determine whether there was an increase or no increase in DMFS score post-RT.

### Tooth decay assessment

A new lesion included new or filled cavities pertaining to root caries or secondary caries, but not incipient caries. No radiographic confirmation was performed in the ORARAD study, at the time points calibrated examiners were recording DMFS values. Tooth decay was measured on all teeth (four surfaces for anterior teeth and five surfaces for posterior teeth) using the decayed, missing and filled surface (DMFS) score on the 128-point scale for 28 teeth present at T0, T6 and T18 [26]. A patient having increased tooth decay post-RT was placed in the DMFS[+] (increase) group. An increase in tooth decay was defined as having at least one additional surface with a positive score for the decayed (D) component of the score. Newly filled teeth surfaces (F) post-RT were used as a surrogate measure of tooth decay, so that patients with at least one newly filled tooth surface post-RT was also placed in the DMFS[+] group. Patients with a ‘decrease’ in DMFS score or no change, were classified in the DMFS[-] (no increase) group. A ‘decrease’ in DMFS, which was recorded for six patients (*i.e*., one patient with 3, two with 2, and three with 1 tooth surface(s)) was interpreted as possible counting or recording discrepancies, between the baseline measurement and T6 or T18. A DMFS[-] patient in the T0 to T6 analysis may become a DMFS[+] patient in the T0 to T18 analysis (*i.e*., T6, T18 DMFS score data).

### Sample collection

Saliva, SSP, buccal mucosa, and tongue samples were collected prospectively at T0, T6 and T18. Saliva was collected while chewing unflavored and unsweetened gum base (The Wrigley Company, Mars, Inc., Chicago, IL). Saliva was initially collected for a 2 min start-up period into a 50 mL conical BD Falcon polypropylene centrifuge tube (Corning, NY). This initial collection of saliva was not used for the determination of SSFR. If collection in the first tube was successful, subjects were directed to continue chewing the gum base and begin saliva collection into a new tube for up to 5 min. The SSFR was determined based on the volume collected in this second tube. The saliva samples of the two tubes were then combined and transferred to a third tube kept on ice for no longer than 30 min before further processing or if not processed, were sent to storage at −80°C.

Subsequently, SSP samples were acquired using OmniSwabs (GE Life Sciences-Buckinghamshire, UK) across the lateral surfaces of all maxillary and mandibular teeth at the junction of the tooth and gingiva. Buccal mucosa bacterial samples were obtained by swabbing both sides of the buccal mucosa for 10 s each. Tongue bacterial samples were obtained by swabbing an approximately 1 cm^2^ region on both sides of the mid dorsal region of the tongue for 5 s.

### Sample processing and microbiome profiling

Saliva samples (1–2 mL) were centrifuged (2,600 × g; 4°C; 15 min) to collect the bacterial pellets. Swab samples were suspended in nuclease-free PBS solution containing 0.04% sodium azide and rotated (2 h at room temperature) to release bacteria from the swab into the solution [27]. The swab sample suspensions were centrifuged (16,000 x g) to harvest bacterial pellets. All pellets were stored at −80°C.

Bacterial genomic DNA from each sample was isolated using a modification of QIAamp DNA mini kit (QIAGEN, Valencia, CA), as previously described [27]. Each sample was processed and analyzed separately for microbiome profiling. HOMI*NGS* was used to identify and semi-quantify bacteria at the species level, as previously described [22]. Briefly, the amplified 16S rRNA gene (V3–V4 region) was sequenced using a modified MiSeq NGS method (Illumina, Inc., San Diego, CA) [28]. Resulting sequence reads were bar-coded and saved electronically.

Oral taxa identification and abundance were determined using the ProbeSeq program, in which sequence reads are first matched against ProbeSeq species probes, in a BLAST-type, electronic hybridization [22]. Sequence reads that had a unique electronic hybridization to one ProbeSeq species probe out of 638 ProbeSeq species probes were counted as one ‘hit’ for that probe. A few species or genera were represented on two different species or genus probes, respectively. Sequence reads not uniquely matched to a species probe were then matched against the 129 ProbeSeq genus probes. Sequences not uniquely matched to a ProbeSeq genus probe were grouped as unmatched reads for that patient sample. The relative abundance of hits by ProbeSeq species and genus probe was determined for each patient sample and used in the statistical analyses.

### Statistical analyses

We first determined overall microbiome changes following cancer therapy (T0 to T6 and T0 to T18) regardless of caries development. In a second step, we identified bacterial profiles potentially promoting tooth decay in those HNC patients who developed tooth decay (DMFS[+] group), compared to those who did not. In a sub-analysis, we determined the microbiome changes for a subset of bacterial species reported to be associated with the development of caries (*i.e*., caries-associated species) (Supplementary Table 3).

Multivariate permutational analysis of variance (PERMANOVA) in PRIMERv7 (PRIMER-E Ltd., Ivybridge, U.K.) was used to compare the beta-diversity between the groups in longitudinal and cross-sectional comparisons (significance level alpha = 0.01). PERMANOVA was performed for paired data from any of the four oral sites (buccal mucosa, SSP, tongue and saliva). We compared microbiome profiles of subjects from T0 to T6 and from T0 to T18 for all patients (Set-1), for patients with common paired oral site samples (Set-2), and for Set-1 patient subsets (A, B, C, D) that had DMFS score data (Supplementary Tables 1 and 2). We implemented a mixed-model PERMANOVA design, unrestricted permutation of raw data, 9,999 permutations, and type III partial sum of squares. In this design, the ‘Time’ factor was fixed (pre- and post-RT) and the random variable ‘Subjects’ was nested into the random variable ‘Treatment’ along with the pre-set fixed variable ‘Oral Site’ (*i.e*., buccal mucosa, SSP, saliva, tongue). ‘Treatment’ was nested in ‘Oral Site’.

To control for the degrees of freedom, ‘Treatment’ was coded as a single factor resulting in eight possible combinations (*i.e*., PERMANOVA ”Treatment” variable levels) for our patient cohort, depending on whether HNC patients received RT with or without CC, IC, or AB treatment (Supplementary Tables 1 and 2). A squared root transformation was applied to relative abundance data for all genus and species probes combined before generation of a Bray-Curtis similarity matrix. A sub-analysis was performed based on species probes corresponding to known caries-associated bacterial taxa only (Supplementary Table 3) [29].

A two-pronged approach was used to compare the microbiome profile changes in the DMFS[+] group compared to those of the DMFS[-] group. First, for HNC patients with DMFS score information available (n = 28 for T0 to T6 and n = 20 for T0 to T18; Supplementary Table 2), we used a cross-sectional PERMANOVA design with ‘Treatment’ as a random factor nested in fixed factors ‘DMFS’ and ‘Oral Site’ to determine whether the profiles of all species and genus probes (n = 767) at T0 were different from the subset of caries-associated probes (n = 45) only.

In a second step, the RA fraction difference (FD) (instead of percentage difference) increase or decrease between T0 and T6 and between T0 and T18 were computed for each probe for all patients (DMFS[+] and DMFS[-] combined). The RA-FD was determined using the formula [(final – initial)/initial], (i) without pseudo-count addition to initial ‘hits’ (infinite initial hits RA-FD outcomes were arbitrarily set to zero, and therefore assumed to be undefined data), or (ii) with addition of a pseudo-count of 0.5 or 1 to initial ‘hits’ (converting infinite initial hits RA-FD outcomes to realistic data while maintaining comparative data relationships) [30,31]. A ‘min-max’ standardization was performed based on the cumulative RA-FD for all 767 probes per patient sample, thereby eliminating negative values and centering the data between 0 and 1. Using this normalized RA-FD data, with or without log(x + 1) transformation, a Kendall’s *tau* dissimilarity matrix was generated using the GrammR package in R v3.4.2 (https://www.R-project.org) [32,33]. The matrix was imported in PRIMERv7 as a ‘dissimilarity’ matrix to perform a PERMANOVA analysis using a design identical to the T0 baseline cross-sectional comparisons noted above.

PERMANOVA Monte-Carlo-corrected p-values were determined for fixed and random factors. Longitudinal analyses with ‘Time’ as the primary factor were performed for all patients (with or without DMFS score information). Cross-sectional baseline along with DMFS[+] *vs*. DMFS[-] RA-FD analyses were performed for the four patient subsets, *i.e*., Subset-A all patients, Subset-B all patients who did not receive IC, Subset-C all patients who did not receive AB, and Subset-D all patients who received CC.

Non-metric multidimensional scaling (nMDS) was used for visual representation of patient sample segregation into clusters. Shannon and Simpson alpha diversity indices for relevant comparisons were determined using PRIMERv7 (PRIMER-E Ltd., Ivybridge, U.K.). Statistical significance (at p < 0.05) of differences between groups for demographic and clinical characteristics and alpha-diversity were determined using appropriate tests (*i.e*., chi-squared, Mann-Whitney U, and Wilcoxon signed-rank tests) in XLSTAT v19.02 (Addinsoft, New York, NY).

## Results

**Figure 1.**
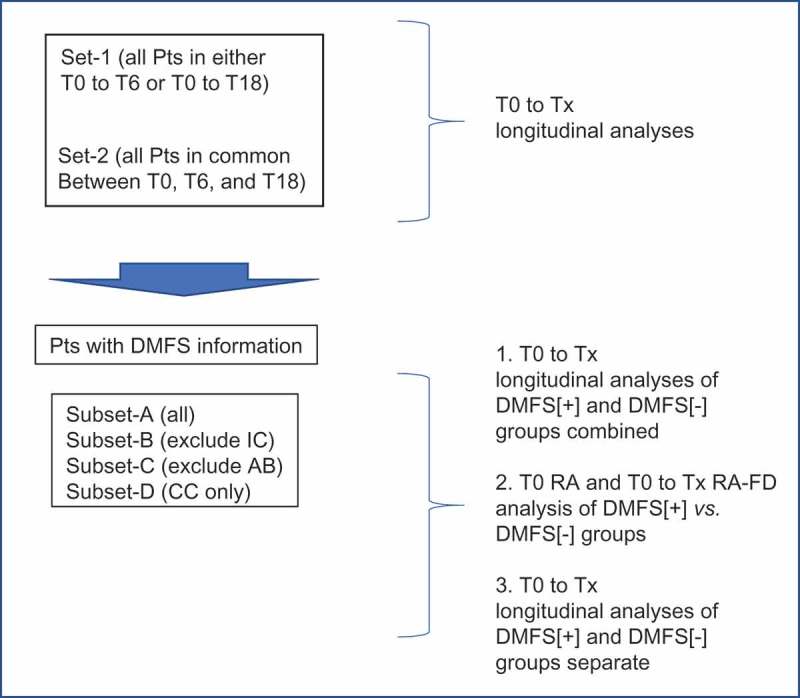
Analytical design for the determination of oral microbiome beta-diversity changes in radiation-treated head and neck cancer patients. Comparisons of beta-diversity were carried out using transformed relative abundances (RA) or standardized RA fraction difference (RA-FD) to determine overall changes following radiation therapy (post-RT), changes occurring in clinically different subgroups, and changes characterizing DMFS[+] and DMFS[-] patients. Pts: HNC patients; T0: baseline time point prior to radiation therapy; Tx: T6 (6-months) or T18 (18-months) post-RT; AB: antibiotics; IC: Induction Chemotherapy; CC: Concurrent Chemotherapy. T0 to Tx corresponds to baseline to 6-months post-RT sampling (T0-T6), or baseline to 18-months post-RT sampling (T0-T18).

A flow chart summarizing the analytical design of the study pertaining to the results described in the following sections is presented in Figure 1.

## Longitudinal microbiome analysis of radiation-treated HNC patients

All longitudinal PERMANOVA analyses showed strong beta-diversity differences between T0 *vs*. T6 and between T0 *vs*. T18 for Sets 1 and 2 and for all Set-1 Subsets A-D (Table 2). Comparisons of T0 to T6 or T0 to T18 included the patients sets ‘all patients’ (Set-1; n = 31) and all patients with oral site types in common with T18 (Set-2; n = 22) with or without DMFS score information (demographics shown in Supplementary Table 1). Comparisons were also performed for patient subsets, all of which had DMFS score information: all patients (Subset-A; n = 28); patients who did not receive IC (Subset-B; n = 20); patients who did not receive AB within 2 weeks of sampling (Subset-C, n = 21); patients who received CC (Subset-D, n = 24) (demographics shown in Supplementary Table 2).

**Table 2.** HNC patients, paired oral samples, and longitudinal PERMANOVA analyses.

*Longitudinal Analysis* *T0-Tx*	Patient Count (M/F)	Patient %M/F	Paired Samples	PERMANOVA Time: All Probes*	PERMANOVA Time: Caries Probes**
Set-1 [T0-T6]	31 (26/5)	84/16	101	0.0001	0.0001
Set-1 [T0-T18]	23 (19/4)	83/17	69	0.0003	0.0001
Set-2 [T0-T6]	22 (18/4)	82/18	60	0.0002	0.0004
Set-2 [T0-T18]	22 (18/4)	82/18	60	0.0005	0.0003
Subset-A [T0-T6]	28 (23/5)	82/18	90	0.0001	0.0001
Subset-A [T0-T18]	20 (16/4)	80/20	58	0.0002	0.0001
Subset-B [T0-T6]	20 (16/4)	80/20	63	0.0001	0.0001
Subset-B [T0-T18]	14 (11/3)	79/21	39	0.0038	0.0033
Subset-C [T0-T6]	21 (17/4)	81/19	66	0.0001	0.0001
Subset-C [T0-T18]	14 (11/3)	79/21	38	0.0017	0.0007
Subset-D [T0-T6]	24 (20/4)	83/17	79	0.0001	0.0001
Subset-D [T0-T18]	19 (15/4)	79/21	55	0.0001	0.0001

Longitudinal multivariate permutational analyses of variance (PERMANOVA) were performed on square root transformed relative abundance data. Monte-Carlo corrected p-values for the fixed variable ‘Time’ are shown with alpha level of significance level set at 0.01. Set-1: all patients with and without DMFS score information Set-2: all patients samples in common across the three timepoints T0, T6, and T18, with and without DMFS score information. Set-1 subsets of patients with DMFS score information: Subset-A (all patients), Subset-B (all patients who did not receive induction chemotherapy), Subset-C (all patients who did not receive antibiotics within 2 weeks of sampling), Subset-D (all patients who received concurrent chemotherapy). T0 to Tx: baseline to 6-months post-RT sampling (T0-T6), or baseline to 18-months post-RT sampling (T0-T18). Gender: M (male), F (female). *Species probes (n = 638) plus genus probes (n = 129); **probes for caries-associated species (n = 45).

**Figure 2.**
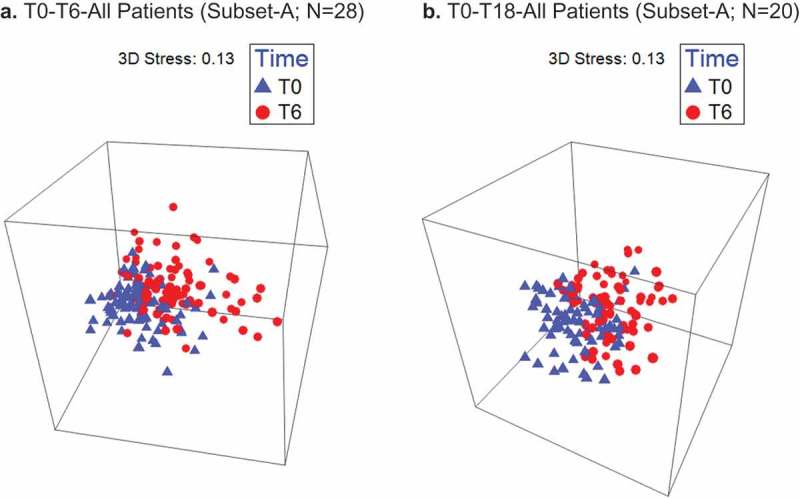
A significant oral microbiome profile shift occurs between T0 and T6 for all RT-treated HNC patients (a) and patients treated with RT and concurrent chemotherapy only (b). Timepoints: T0-pre-RT baseline sampling, T6-post-RT sampling at 6 months.

**Figure 3.**
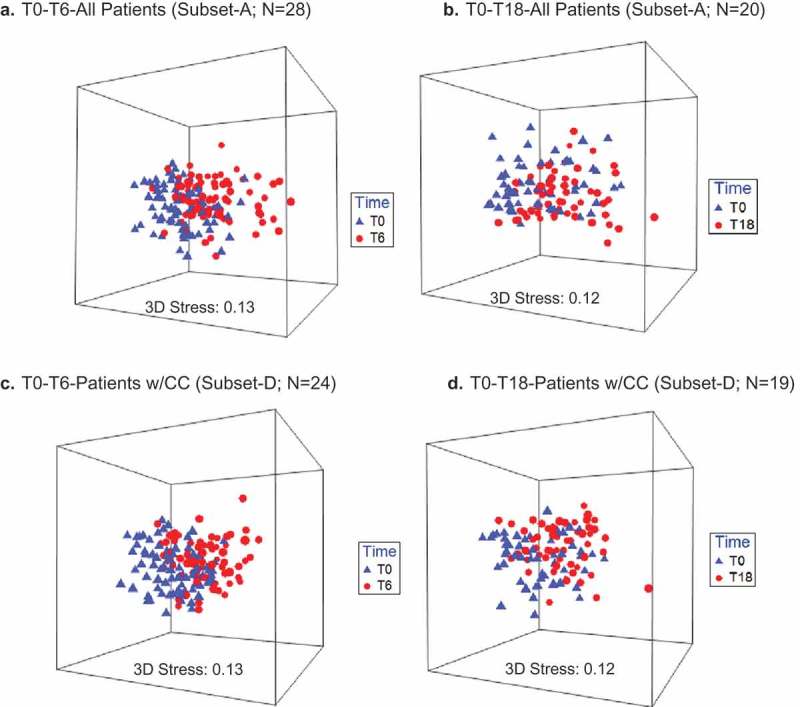
Non-metric multidimensional scaling (nMDS) of caries-associated species profiles of oral sites for all patients and patients who underwent concurrent chemotherapy (CC) T0 to Tx. **a**. T0-T6-All Patients (Subset-A; n = 28). **b**. T0-T18-All Patients (Subset-A; n = 20). **c**. T0-T6-Patients w/CC (Subset-D; n = 24). **d**. T0-T18-Patients w/CC (Subset-D; n = 19). A significant shift of caries-associated species profiles occurs between T0 to T6 (a, c), and T0 to T18 (b, d) for RT-treated HNC patients and for patients treated with RT and concurrent chemotherapy. T0 to Tx corresponds to baseline to 6-months post-RT sampling (T0-T6), or baseline to 18-months post-RT sampling (T0-T18).

Figure 2(a,b) 3D-nMDS show T0 to T6 clustering of Subset-A (n = 28) and Subset-D (**n**= 24) patients’ samples. A list of 45 ‘caries-associated’ species probes and 14 ‘health-associated’ species probes, established based on Tanner et al. [29] and other references, are shown in Supplementary Table 3. The beta-diversity of the caries-associated species in the T0 to T6 and T0 to T18 analyses were also significant for all groups (Table 1, Figure 3(a,b,c,d)).

Prior to conducting PERMANOVA analyses, examination of the HOMI*NGS* sequence reads detection data was performed. Descriptive HOMI*NGS* sequence reads detection data for Subset-A are summarized in Supplementary Table 4. Unmatched reads as a percent of total reads increased slightly post-RT. Although three patients of Set-1 (n = 31) did not have DMFS score information, removing those from the analysis (Subset-A, n = 28) did not significantly affect demographic and clinical characteristics (data not shown).

Analysis of the sequence reads showed that detection of species at timepoints T0, T6 and T18 for Subset A (all patients) was positive for 485, 481 and 484 out of the 634 ProbeSeq species probes and detection of genera was positive for 102, 105, 102 out of the 129 ProbeSeq genus probes, respectively. The approximate average numbers of species detected per patient sample were 113, 113, 122 for timepoints T0, T6 and T18, respectively, while the numbers of genera per patient sample detected with genus probes were about 37, 38 and 40, respectively. Mean and median total numbers of reads per patient were similar for T0 *vs*. T6 and T0 *vs*. T18 (Supplementary Table 4). Overall, detection data were equally distributed across time points.

In addition, a total of 101 paired oral samples were processed for Set-1 patients (n = 31) instead of a maximum of 124 (4 × 31), still providing enough power for analysis by including the data for all oral sites in the PERMANOVA analysis. Also, the results did not appear to depend on the fact that patients and paired samples ranging from 1–4 oral sites per patient may not have been all in common between T0 to T6 and T0 to T18 comparing Set-1 to Set-2 (Table 2). We further performed single site (*e.g*., SSP alone) or two-site (*e.g*., SSP and saliva) PERMANOVA analyses, independently, but these did not return statistically significant p-values of < 0.05 (data not shown).

1. Cross-sectional baseline and pre- to post- RT comparison of microbiome relative abundance fraction difference (RA-FD) between DMFS[+] and DMFS[-] patients

Comparing the DMFS[+] patient group to the DMFS[-] group, there were no statistically significant baseline (T0) differences in average DMFS score or SSFR, or average percentage difference in the T0 to T6 or T0 to T18 reduction of SSFR, as shown in Table 1 for Subset-A and Supplementary Table 2 for Subsets A-D. However, for both DMFS[+] and DMFS[-] groups, there was a SSFR reduction at T6 and T18 post-RT. Also, there were no significant differences in the representation of oral sites (data not shown) or differences in average total radiation dose received (Table 1) when comparing DMFS[+] to DMFS[-] patient groups.

We found similar differences in relative abundance changes in DMFS[+] (RT caries affected) compared to DMFS[-] (RT caries unaffected) patients for Subsets-A and D in the T0 to T6 and T0 to T18 comparisons for all probes and for the caries-associated species probes, based on the minmax standardized and/or log(x + 1) transformed RA-FD *tau* dissimilarity matrix (PERMANOVA, p < 0.01) (Table 3). The results for Subsets-A and D were also similar in the baseline comparisons (Table 3). The mean SSFR reduction differences between DMFS[+] and DMFS[-] groups for Subsets A and D were not statistically significant, whether in the T0 to T6 or T0 to T18 comparison (see grey highlighted row in Supplementary Table 2). A graphical nMDS representation, based on RA-FD *tau*-dissimilarity, showing the separation of Subset-D patient samples in the comparison of DMFS[+] *vs*. DMFS[-] groups using all probes is presented in the Supplementary Figure.

**Table 3.** DMFS[+] and DMFS[-] HNC patients and cross-sectional PERMANOVA analyses.

Subsets T0-Tx	Data transformation	Pt Count DMFS[+]	Pt Count DMFS[-]	Total Pts	Cross-sectional RA-FD Analysis DMFS [+] *vs*. DMFS[-]		Baseline Analysis DMFS[+] *vs*. DMFS[-]	
					All Probes*	Caries Probes**	All Probes	Caries Probes
Subset-A	**Original Data**	11	17	28	0.0006	0.0095	0.0206	0.1412
[T0-T6]	**Add 1 Hit**				0.0002	0.0007		
	**Add 0.5 Hit**				0.0003	0.0008		
	**Original Data^log^**				0.0006	0.0096		
	**Add 1 Hit^log^**				0.0003	0.0005		
	**Add 0.5 Hit^log^**				0.0003	0.0006		
Subset-A	**Original Data**	7	13	20	0.0210	0.2662	0.0006	0.0039
[T0-T18]	**Add 1 Hit**				0.0346	0.3672		
	**Add 0.5 Hit**				0.0354	0.3658		
	**No added Hit^log^**				0.0215	0.2634		
	**Add 1 Hit^log^**				0.0362	0.3728		
	**Add 0.5 Hit^log^**				0.0380	0.3639		
Subset-B	**Original Data**	6	14	20	0.0737	0.0502	0.0021	0.0243
[T0-T6]	**Add 1 Hit**				0.0687	0.0653		
	**Add 0.5 Hit**				0.0631	0.0534		
	**Original Data^log^**				0.0713	0.0519		
	**Add 1 Hit^log^**				0.0680	0.0655		
	**Add 0.5 Hit^log^**				0.0693	0.0540		
Subset-B	**Original Data**	11	3	14	0.3397	0.3834	0.0501	0.0919
[T0-T18]	**Add 1 Hit**				0.2135	0.4392		
	**Add 0.5 Hit**				0.4025	0.4275		
	**Original Data^log^**				0.3459	0.3773		
	**Add 1 Hit^log^**				0.2101	0.4385		
	**Add 0.5 Hit^log^**				0.3937	0.4321		
Subset-C	**Original Data**	8	13	21	0.0159	0.0567	0.0278	0.0388
[T0-T6]	**Add 1 Hit**				0.0076	0.0130		
	**Add 0.5 Hit**				0.0099	0.0099		
	**Original Data^log^**				0.0158	0.0565		
	**Add 1 Hit^log^**				0.0076	0.0129		
	**Add 0.5 Hit^log^**				0.0079	0.0117		
Subset-C	**Original Data**	11	3	14	0.1370	0.2628	0.0907	0.1106
[T0-T18]	**Add 1 Hit**				0.0338	0.2485		
	**Add 0.5 Hit**				0.0351	0.2509		
	**Original Data^log^**				0.1372	0.2600		
	**Add 1 Hit^log^**				0.0345	0.2469		
	**Add 0.5 Hit^log^**				0.0334	0.2534		
Subset-D	**Original Data**	11	13	24	0.0005	0.0042	0.0295	0.1075
[T0-T6]	**Add 1 Hit**				0.0002	0.0001		
	**Add 0.5 Hit**				0.0001	0.0003		
	**Original Data^log^**				0.0008	0.0029		
	**Add 1 Hit^log^**				0.0001	0.0001		
	**Add 0.5 Hit^log^**				0.0001	0.0004		
Subset-D	**Original Data**	7	12	19	0.0200	0.2728	0.0002	0.0001
[T0-T18]	**Add 1 Hit**				0.0488	0.1433		
	**Add 0.5 Hit**				0.0449	0.1442		
	**Original Data^log^**				0.0198	0.2693		
	**Add 1 Hit^log^**				0.0474	0.1409		
	**Add 0.5 Hit^log^**				0.0448	0.1445		

Cross-sectional ‘differential’ multivariate permutational analysis of variance (PERMANOVA) analyses are represented in the ‘Cross-sectional RA-FD Analysis’ column. Pseudo-counts of 1 and 0.5 hits were added to original raw abundance data. Relative abundance fractional differences (RA-FD) were determined followed by min-max standardization for all probes (n = 767, ‘All Probes’ column) and separately for the caries-associated probes (n = 45, ‘Caries Probes’ column), for all subsets (A-D) and for all time period comparisons (T0-Tx) by group (DMFS[+] vs. DMFS[-]). A *tau* dissimilarity matrix was then generated using GrammR in R for each analysis. PERMANOVA Monte-Carlo corrected p-values for the fixed variable ‘DMFS Group’ were determined based on the *tau* dissimilarity matrices. This process was repeated after applying log_10_(x + 1) (‘log’ superscript in table) transformation to the min-max standardized values. Cross-sectional baseline PERMANOVA analyses (Baseline Analysis column) were also performed on square root transformed relative abundance data (without pseudo-count additions). Monte-Carlo corrected p-values for the fixed variable ‘DMFS Group’ are shown. The alpha significance level was set at 0.01. Set-1 subsets of patients (Pts) with DMFS information were: Subset-A, all patients; Subset-B, all patients who did not receive induction chemotherapy; Subset-C, all patients who did not receive antibiotics; Subset-D, all patients who received concurrent chemotherapy. Pt count: Number of patients; T0: baseline sampling; T0 to Tx: T0 to 6 months post-RT and T0 to 18 months post-RT sampling. *Species probes (n = 638) and genus probes (n = 129) **Probes for caries-associated species (n = 45).

In addition, for the largest subset (Subset-A [T0-T6]; n = 28), there were 7.82% (5,401 out of 69,030) data point instances where RA-FD was arbitrarily set to zero due to an ‘initial’ zero count, which did not impact the results as compared to the pseudo-count results (Table 3). Using the relative abundance magnitude difference (final RA – initial RA) also did not affect the results (data not shown).

There was no significant baseline microbiome beta-diversity difference based on the Bray-Curtis similarity metric in the T0 to T6 comparison for Subsets-A and D (all patients and patients receiving CC) (Table 3). On the other hand, there was no RA-FD *tau* dissimilarity difference between the DMFS[+] and DMFS[-] patient groups for Subsets-A and D in the T0 to T18 comparison while taking into account DMFS score changes at T6. However, there was a significant difference at baseline in beta-diversity based on the Bray-Curtis similarity metric (Table 3). The baseline DMFS[+] *vs*. DMFS[-] group comparison of T0 to T6 did not include the same patients as the T0 to T18 comparison due to the possible increase in DMFS score recorded at T18 for some patients. Such change may have generated a T18 DMFS[-] group as being least likely to develop caries, contrasting with the T18 DMFS[+] group which included patients who were initially classified in the T6 DMFS[-] group. In addition, whether the RA-FD minmax transformation for caries-associated species was performed based on all 767 probes or only on the 45 caries-associated species subcommunity, did not affect the results (Table 3). This suggests a global coherent community effect.

**Figure 4.**
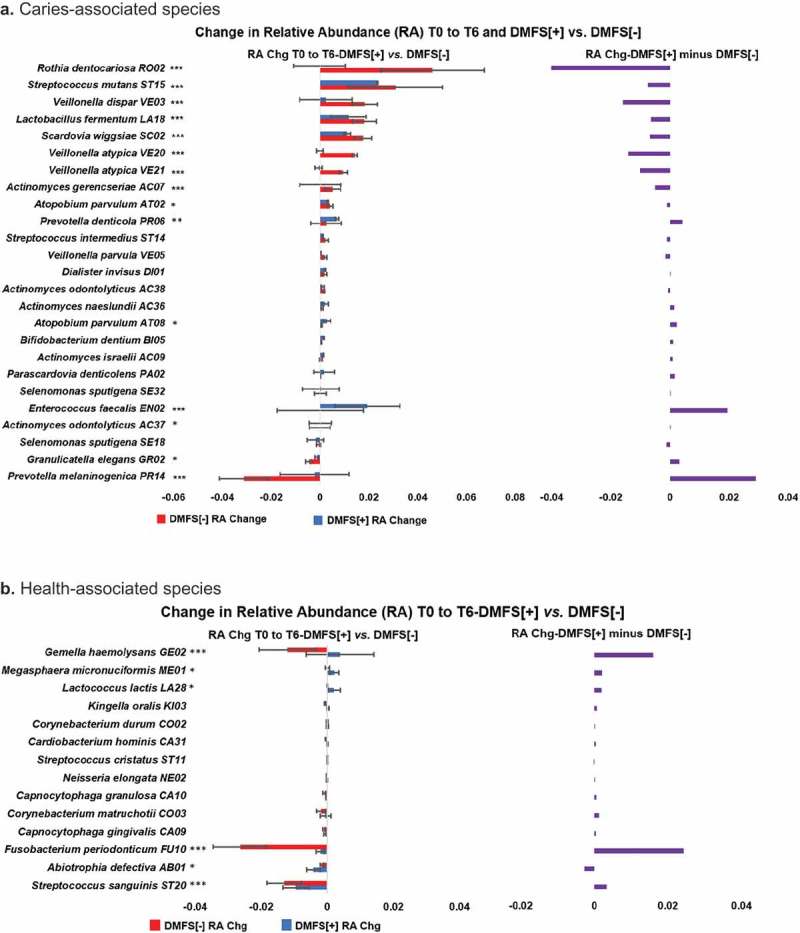
Changes in relative abundance of caries- and health- associated species in DMFS[+] compared to DMFS[-] RT-treated HNC patients (Subset-D, all patients with CC). **a**. Caries-associated species. **b**. Health-associated species On the left of each chart, the T0 to T6 relative abundance (RA) change (Chg), *i.e*., T6 average RA minus T0 average RA by species is shown for the DMFS[+] and DMFS[-] groups of Subset-D, all HNC patients treated with RT and concurrent chemotherapy, for caries- (**a**) and health- (**b**) associated species. The difference in the group RA’s shown on the left, *i.e*., DMFS[+] average RA minus DMFS[-] average RA, by species is presented on the right of the charts. HOMI*NGS* species probes identification 4-character codes are shown. A chi-squared test was used to determine the significance of differences in RA increases/decreases between the DMFS[+] and DMFS[-] groups. *p < 0.05; **p < 0.01; ***p < 0.001.

Significant differences between DMFS[+] and DMFS[-] groups were observed in all oral sites combined. Changes in relative abundance of the 45 caries-associated species and the 14 health-associated species (Supplementary Table 3) for T0 to T6 in the DMFS[+] *vs*. DMFS[-] patient groups of Subset-D are presented in Figure 4. As seen in Figure 3a, in DMFS[-] group *Rothia dentocariosa, Streptococcus mutans, Veillonella dispar, Lactobacillus fermentum, Scardovia wiggsiae, Veillonella atypica, Actinomyces gerencseriae* and *Atopobium parvulum* (in decreasing rank order) had the largest increase in mean relative abundance. Furthermore, in this patient group, *R. dentocariosa, V. atypica* and *V. dispar* (with a T0 mean RA of 3.03/0.74/1.18%, respectively) increased in mean RA by 4.63/2.37/2%, respectively at T6. In the DMFS[+] group, however, these three species showed very small change, *i.e*., −0.01/-0.03/0.12%, respectively. Meanwhile, a significant increase in mean relative abundance was observed in both the DMFS[+] and DMFS[-] groups for *S. mutans, L. fermentum*, and *S. wiggsiae* (DMFS[+] group: T0: 1.79/0.04/0.02%, respectively;T6: 2.95/0.62/0.57%, respectively; DMFS[-] group: T0: 0.27/0.02/0.02%, respectively;T6: 3.37/1.84/1.80%, respectively) (Figure 4a). We also noted a high increase in relative abundance of *Enterococcus faecalis* in a single DMFS[+] patient and a significant decrease in mean relative abundance of *Prevotella melaninogenica* in the DMFS[-] group (Figure 4a). For the caries-associated species *S. mutans* and *P. melaninogenica*, the largest contribution to the overall ‘all sites’ differences were observed in tongue (largest difference) and SSP samples.

Most prominent T0 to T6 changes in mean relative abundance for health-associated species, were observed for *Gemella haemolysans, Fusobacterium periondonticum, Abiotrophia defectiva*, and *Streptococcus sanguinis*, showing a reduction in the DMFS[-] group (Figure 4b). While the mean relative abundance of *G. haemolysans, Megasphaera micronuciformis* and *Lactococcus lactis* did increase in the DMFS[+] group, only *A. defectiva* showed a larger mean relative abundance decrease than for the DMFS[-] group (Figure 4b). For the health-associated species *S. sanguinis, G. haemolysans* and *F. periodonticum*, the largest contribution to the overall ‘all sites’ differences were observed in SSP, buccal mucosa and tongue samples, respectively.

Changes in alpha-diversity (Shannon and Simpson indices) for longitudinal comparisons were not significantly different for the all probe analyses (significance level alpha = 0.01), while most comparisons with caries-associated probes showed significance, including for Subset-D (all receiving CC) (Supplementary Table 5).

## Discussion

In this study, we show that the beta-diversity between RT-treated HNC patients who experienced an increase in DMFS score (DMFS[+] group) and those who did not (DMFS[-] group), for the four Set-1 subsets of patients (A-D), differed in regard to co-treatment with IC, AB and CC (Table 2).

The PERMANOVA results with Set-2 were consistent with Set-1 and Subset-A (Table 2).

There were no significant baseline (T0) differences in DMFS score or SSFR between the DMFS[+] and DMFS[-] patient groups. In addition, there were no significant differences in the SSFR reductions from T0 to T6 and from T0 to T18, for any of the subsets (A-D).

The cross-sectional RA-FD DMFS[+] *vs*. DMFS [-] group analysis also showed that antibiotics and induction chemotherapy had a strong effect on microbiome changes regardless of whether all probes (n = 767) or only the caries-associated species probes (n = 45) were used in the analysis (Table 3).

In the baseline (T0) cross-sectional DMFS[+] *vs*. DMFS[-] RA-FD analyses of Subset-A and Subset-D, there was no difference in Bray-Curtis beta-diversity, while there was a difference between the T0 to T6 overall standardized and/or transformed RA-FD (for the ‘all probes’ and for the caries-associated species) (Table 3). In contrast, for T0 to T18, the opposite result was obtained. That said, for Subsets A and D, 19 patients were in common between the T0 to T6 and T0 to T18 comparisons. This paradox can be explained by the possibility that the T0 to T6 changes (‘short term’ effects) are mainly due to cancer treatment effects, while the T0 to T18 changes are more dependent on the baseline microbiome and a ‘normal’ host-response status (‘long-term’ effects). One could argue that the difference in baseline beta-diversity status could be due to the reduction in sample size and DMFS[+] *vs*. DMFS[-] proportion differences (Supplementary Tables 1 and 2), although a patient originally classified in DMFS[+] group for T0 to T6 was also classified as DMFS[+] for T0 to T18. However, a longitudinal sub-analysis of separate DMFS[+] and DMFS[-] groups for the four subsets A-D was performed showing that there were beta-diversity changes for both DMFS groups in the T0 to T6 comparisons but not for the DMFS[-] group in the T0 to T18 comparisons (Table 4).

**Table 4.** Longitudinal T0-Tx sub-analysis of separate DMFS[+] and DMFS[-] groups.

DMFS+	PERMANOVA Time: All Probes*	PERMANOVA Time: Caries Probes**
Subset-A [T0-T6]	0.0001	0.0001
Subset-A [T0-T18]	0.0001	0.0006
Subset-B [T0-T6]	0.0056	0.0084
Subset-B [T0-T18]	0.0015	0.0030
Subset-C [T0-T6]	0.0091	0.0030
Subset-C [T0-T18]	0.0077	0.0052
Subset-D [T0-T6]	0.0001	0.0001
Subset-D [T0-T18]	0.0002	0.0002
DMFS-	PERMANOVA Time: All Probes*	PERMANOVA Time: Caries Probes**
Subset-A [T0-T6]	0.0001	0.0001
Subset-A [T0-T18]	0.0404	0.0842
Subset-B [T0-T6]	0.0001	0.0001
Subset-B [T0-T18]	0.1120	0.1397
Subset-C [T0-T6]	0.0001	0.0001
Subset-C [T0-T18]	0.2105	0.2592
Subset-D [T0-T6]	0.0001	0.0001
Subset-D [T0-T18]	0.0295	0.0177

Longitudinal PERMANOVA analyses were performed on square root transformed relative abundance data. T0 to Tx corresponds to baseline to 6-months post-RT sampling (T0-T6), or baseline to 18-months post-RT sampling (T0-T18). Monte-Carlo corrected p-values for the fixed variable ‘Time’ are shown with alpha significance level of 0.01. T0: baseline sampling; T6: sampling at 6 months post-RT; T18: sampling at 18 months post-RT. Set-1 subsets of patients with DMFS information were: Subset-A: all patients; Subset-B: all patients who did not receive induction chemotherapy; Subset-C: all patients who did not receive antibiotics; Subset-D: all patients who received concurrent chemotherapy. *Species probes (n = 638) and genus probes (n = 129) (*i.e*., total of 767 probes) **Probes for caries-associated species (n = 45).

These results suggest a better recovery to a ‘T0 baseline’ microbiome status for these patients who may have had an SCC-associated oral microbiome at baseline. One possible explanation for these results is that the oral microbiome and its interaction with the host govern such recovery, in conjunction with the effects of other factors, and represent an important determinant of caries development.

Overall, our RA-FD approach using Kendall’s *tau* as a distance metric in GrammR [32,33] produced consistent and biologically meaningful results (Table 2). Although, we recognize that many methods for microbiome counts data analyses have been recently developed, there is currently little consensus in the field about their implementation [30,31,34,35].

Surprisingly, normally abundant caries-associated species which substantially increased in relative abundance in the DMFS[-] group of Subset-D (*i. e., S. mutans, L. fermentum, S. wiggsiae*, and *R. dentocariosa*) did not increase to the same extent in the DMFS[+] group (Figure 4a). However, a large decrease of *P. melaninogenica* relative abundance was observed in the DMFS[-] group of Subset-D, which was on average about 14 times larger than the *P. melaninogenica* relative abundance decrease noted in the DMFS[+] group (p < 0.001) (Figure 4a). The relative abundance of this species decreased in five of the 11 DMFS[+] patients compared to decreasing in 11 of the13 DMFS[-] patients (p < 0.05).

*P. melaninogenica* has been associated with severe early childhood caries (S-ECC) [36,37]. In a study by Agnello et al. [37], children with ≥ 2% relative abundance of *S. mutans* were 11 times more likely to be in the S-ECC group, while those with ≥ 2% *P. melaninogenica* were eight times more likely to be in the S-ECC group [38]. Similar to S-ECC, radiation caries can form on the smooth surfaces of the incisors, premolars and molars, which are generally most resistant to decay [14,39]. However, it remains to be elucidated how a reduction of abundance of *P. melaninogenica* in dental plaque would reduce the susceptibility to smooth surfaces caries of radiation-treated HNC patients, including those who experience an increase in *S. mutans*. We also found that the relative abundance of the health-associated species *A. defectiva* was significantly decreased in the DFMS[+] group compared to DMFS[-] (Figure 4b). However, there were no significant DMFS[+] *vs*. DMFS[-] differences for other health-associated species such as *Corynebacterium durum* and *Corynebacterium matruchotii* (Figure 4b) [40]. Moreover, with *G. haemolysans* and *F. periodonticum*, the relative abundance was significantly decreased in the DFMS[-] group to a large extent compared to the other 12 health-associated species (Figure 4b). In this respect, the association of these species with ‘health’ does not necessarily mean inducing ‘health’, as these species may have simply been found to be more abundant in control subjects having a healthy oral cavity. Overall, however, while there were large increases in the relative abundance for several caries-associated species post-RT in the DMFS[+] group (Figure 4a), only small changes were observed for the health-associated species (Figure 4b).

In future studies, it will be of interest to recruit a larger number of HNC patients to better determine the extent to which induction chemotherapy, antibiotics, concurrent chemotherapy, and other factors such as oral hygiene, impact the development of caries using multivariate regression analyses [41]. Moreover, current improved metagenomic approaches could allow us to address strain level differences or novel species discovery [42,43]. Finally, microbiome metatranscriptomic/metabolomic approaches could be used to determine how changes in the relative abundance may correlate with changes in activity that affect the host response [44,45].

## Conclusions

Our data suggest that baseline microbiome difference is an important factor that can explain dental caries outcome in radiation-treated HNC patients. Our data suggest a cariogenic role of *P. melaninogenica* and a potential protective role of certain bacterial species such as *A. defectiva*, however more detailed studies are necessary for confirmation. Accordingly, it appears critical to understand how differences in microbiome profiles at baseline in HNC patients undergoing cancer treatment could result from host-defense weaknesses which may be enhanced by non-compliance with good oral hygiene practices. In particular, the role of protective defensins and host genes governing interactions within the oral microbial sub-communities remains to be elucidated [46,47].

## Data Availability

Clinical and HOMI*NGS* metadata files are available as supplementary material Supplemental files 7-10.
