# Supplementary material for: Caries-associated oral microbiome in head and neck cancer radiation patients: a longitudinal study
Source: J Oral Microbiol. 2019 Mar 8;11(1):1586421. doi: 10.1080/20002297.2019.1586421 (PMC6419625; doi:10.1080/20002297.2019.1586421)
Supplement: Supplemental Material [file ZJOM_A_1586421_SM8420.zip › supplemental data/Supplemental_file_5_JOM_02_07_2019.pdf]

Supplementary Figure. Non-metric multidimensional scaling of oral samples DMFS[+] *vs.* DMFS[-] patients of Subgroup-D (concurrent chemotherapy; N=24) T0 to T6 based on fold change *tau* dissimilarity matrix (all probes)

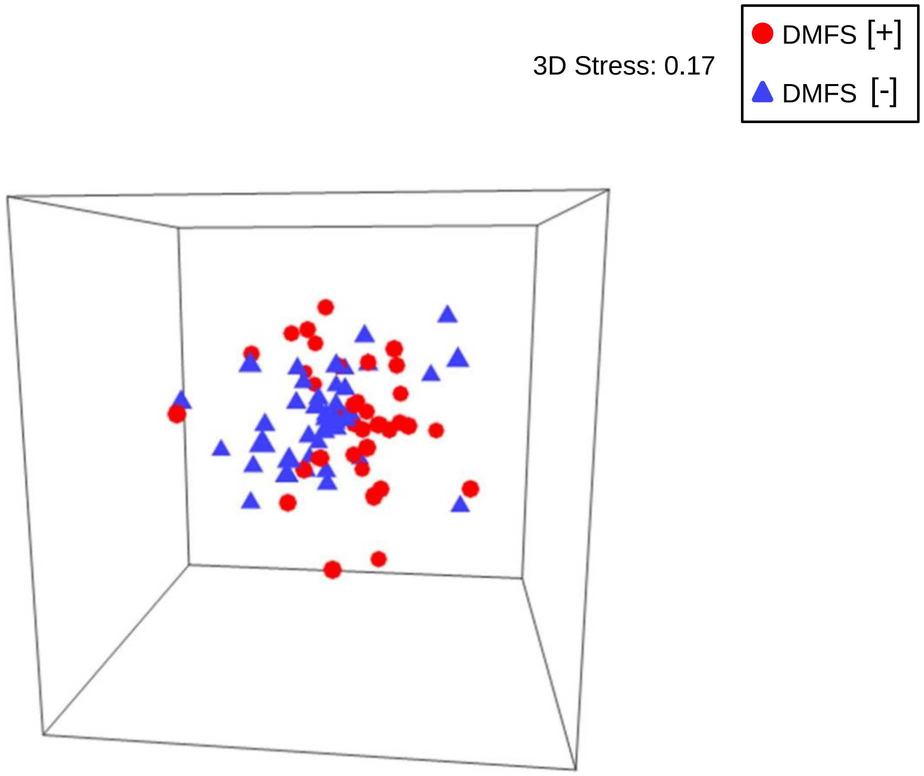

A significant shift of microbiome profiles occurs between T0 and T6 for DMFS[+] compared to DMFS[-] HNC-treated patients.

NI and I are “no increase” and “increase” in DMFS, respectively.
